# Fabrication and Characterization of Carboxylated Lignin Sulfonate Modified Epoxidized Soybean Oil Wood Adhesive Cured by Maleic Anhydride

**DOI:** 10.3390/polym18172048

**Published:** 2026-08-24

**Authors:** Liping An, Zhigang Liu, Xinran Li

**Affiliations:** 1 College of Science, Inner Mongolia Agricultural University, Hohhot 010018, China; shuijing_alp@126.com (L.A.);; 2 Inner Mongolia Key Laboratory of Soil Quality and Nutrient Resource, Hohhot 010018, China; 3 College of Material Science and Art Design, Inner Mongolia Agricultural University, Hohhot 010018, China

**Keywords:** epoxidized soybean oil, maleic anhydride, carboxylated lignin sulfonate, wood adhesive

## Abstract

In this work, a formaldehyde-free bio-based wood adhesive was successfully fabricated using epoxidized soybean oil (ESO) cross-linked with maleic anhydride (MA) and carboxylated lignin sulfonate (CLS). The effect of CLS substitution dosage on the bonding performance was systematically investigated. The results indicated that the dry shear strength and wet shear strength of the adhesive exhibited a typical non-monotonic variation with increasing CLS content, reaching the maximum values of 1.79 MPa and 1.38 MPa, respectively, at a CLS substitution ratio of 40 mol% relative to MA. These mechanical properties fully meet and exceed the requirements of the Chinese national standard for wood adhesives. Orthogonal experiments were further conducted to optimize the hot-pressing process parameters and the optimal conditions were determined as follows: hot-pressing temperature of 130 °C, pressing time of 10 min, glue spread of 280 g/m^2^, and pre-mixing time of 70 min. FTIR, DSC, and TGA characterizations confirmed the complete curing reaction of the adhesive system. The introduced CLS served as both a reactive curing agent and an efficient catalytic component, which effectively reduced the curing temperature, while the incorporation of MA significantly improved the thermal stability of the cured adhesive. This study provides a feasible strategy for the preparation of high-performance, low-cost, and environmentally friendly bio-based wood adhesives.

## 1. Introduction

Adhesives are used in large quantities in the wood industry, with aldehyde-containing adhesives accounting for more than 90% of the wood adhesive market [1]. However, aldehyde-containing adhesives are non-renewable resources, and they release harmful gases during use and for a period of time after bonding, which affects human health [2]. Therefore, bio-based environmentally friendly adhesives have become a hot research topic.

Lignin, a polymer based on phenylpropane derivatives, is a widely available renewable raw material on Earth. Due to its complex macromolecular structure, it is difficult to be utilized directly. Thus, as a waste product of the paper industry, it is used as a fuel or discarded [3]. The renewable, inexpensive, non-toxic, and non-hazardous nature of lignin has attracted the interest of a large number of researchers through various modification methods in order to enhance its use [4,5,6,7,8]. As a renewable polymer with a large number of phenolic hydroxyl groups, lignin has an attractive potential as an adhesive which can be blended with phenolic resin to reduce the amount of phenol used, as well as adsorbing formaldehyde to reduce formaldehyde release [9,10,11]. However, most of the current studies on lignin adhesives focus on the preparation of plywood after blending with phenolic resin, which cannot fundamentally solve the problem of formaldehyde release.

Soybean oil consists of four fatty acids, linoleic acid, oleic acid, palmitic acid, and linolenic acid, and each soybean oil molecule has an average of 4.6 double bonds [12,13]. Epoxidation reagents can react with the unsaturated double bonds in soybean oil to produce epoxidized soybean oil (ESO) [14,15]. ESO has a wide range of applications and is used in plastic plasticizers, coatings, composites, adhesives, etc. ESO contains epoxy groups, and thus ESO can be cured by dicarboxylic acids, anhydrides, and diamines for the preparation of thermosetting epoxy resin materials, but the curing time is too long, usually taking several hours or even longer [16,17,18,19,20]. In addition, some researchers have used ESO to prepare bio-based adhesives. In order to convert ESO into high-performance wood adhesives, some studies have chemically modified ESO [21,22], but the complex modification increases the difficulty of adhesive preparation. Some other studies mixed ESO with aldehyde-containing adhesives [23,24], but formaldehyde was still released. Traditional petroleum-based curing agents are non-renewable and environmentally unfriendly. To improve the bio-based content and sustainability of ESO-based adhesives, partial maleic anhydride was replaced by renewable carboxylated lignin sulfonate. CLS can simultaneously participate in cross-linking reaction and catalyze the curing process, which is beneficial to energy-saving curing and green preparation of bio-based adhesives [25]. Sun et al. found that carboxylated lignin can cure bisphenol A epoxy resin, and make the curing time and temperature of bisphenol A epoxy resin decrease [26].

Numerous previous investigations have focused on epoxidized soybean oil (ESO) modified with raw lignin or unmodified lignosulfonate [27]. Nevertheless, pristine lignin contains very few reactive carboxyl groups, which makes it serve only as an inert physical filler instead of an active cross-linker. This deficiency causes low crosslink density and demands a relatively high curing temperature during preparation. In addition, widely reported ESO–acrylate adhesives are cured through free-radical polymerization of carbon-carbon double bonds, which yields brittle cured networks accompanied by unsatisfactory hydrothermal and water-resistant properties [28,29].

Distinct from these two typical systems, carboxylated lignin sulfonate (CLS) was fabricated through chemical carboxylation modification in the present study. CLS can not only take part in ring-opening esterification crosslinking with epoxy groups of ESO, but also catalyze the curing reaction between maleic anhydride (MA) and ESO. Replacing partial petroleum-derived MA with renewable CLS effectively cuts down the dosage of non-renewable curing agents. Compared with simple ESO-lignin mixtures, the optimized CLS/MA co-curing system alleviates the serious agglomeration of lignin fractions and attains much higher bonding shear strength. Unlike ESO–acrylate adhesives, the three-dimensional ester cross-linked network provides the adhesive with preferable mechanical toughness and outstanding water resistance. Moreover, the dual role of CLS (crosslinking participant and catalyst) has been comprehensively validated by FTIR, DSC, and TGA characterizations. This study supplements fundamental research regarding lignin-modified ESO bio-based wood adhesives.

## 2. Experimental Section

### 2.1. Materials

Epoxidized soybean oil (ESO, purity > 96%, accurate epoxy value = 6.2 mol/100 g) was purchased from Aladdin Reagent Co., Ltd. (Shanghai, China) and used without further purification. Sodium chloroacetate (≥99%) and maleic anhydride (≥98%) were procured from Shanghai McLean Biochemical Science and Technology Co., Ltd. (Shanghai, China). Zinc acetylacetonate (≥95%) was obtained from Beijing Myriad Science and Technology Co., Ltd. (Beijing, China), while sodium lignosulphonate (average Mw ~52,000, average Mn ~7000)) was acquired from Hefei BASF Bio-technology Co., Ltd. (Hefei, China). A kind of poplar veneer was purchased from Guangzhou, China, with dimensions of 300 × 150 × 2 mm and a moisture content of 8%.

### 2.2. Preparation of Carboxylated Lignin Sulfonate (CLS)

To begin, 10 g of sodium lignosulfonate, 2 g of sodium chloroacetate, and 30 mL of distilled water were added sequentially to the flask and stirred at room temperature until fully dissolved. The solution was then adjusted to a pH of 11 using sodium hydroxide, and the reaction was carried out in a water bath at 80 °C for 1.5 h; during the reaction, the pH had to be carefully maintained at around 11. After the reaction was completed, the solution was cooled to room temperature. Then the pH was adjusted to 1 with sulfuric acid, and the resulting precipitate was obtained via centrifugation. The crude solution was placed in a dialysis bag with a molecular weight cut-off of 3500 Da and dialyzed for 48 h to remove residual low-molecular-weight salts and unreacted reagents. During dialysis, deionized water was replaced every 6 h to ensure thorough purification. The purified sample was freeze-dried for 12 h to obtain the final product. Acetic acid was eluted using calcium acetate ion exchange, and the carboxyl group content was then quantitatively determined using an alkaline solution. The carboxyl group content of CLS was determined to be 2.4 mmol·g^−1^ [30].

### 2.3. Design of Controlled Experiment

To prepare different adhesive samples, carboxylated lignin sulfonate (CLS) was first dispersed in distilled water and stirred at 100 °C and 150 r/min for 3 min. Subsequently, epoxidized soybean oil (ESO) was incorporated with adequate stirring. Afterwards, a prescribed dosage of maleic anhydride (MA) was added and the blend was stirred continuously at 100 °C and 150 r/min for 70 min. Detailed adhesive formulations are summarized in Table 1. The substitution ratio of CLS was calculated based on the molar ratio of carboxyl groups in CLS to those in MA.

The solid content, viscosity, and pH of as-prepared adhesives were characterized referring to GB/T 14074-2017. Kinematic viscosity was measured at a constant temperature of 23 ± 1 °C using an SYD-03 kinematic viscosity tester (Dongfang Wei’an Technology Co., Ltd., Jilin, China). The pH values were determined under a controlled temperature of 25 ± 1 °C with a Starter 2100 pH meter (Haus Instruments (Shanghai) Co., Ltd., Shanghai, China).

### 2.4. Design of Orthogonal Experiment

The optimal adhesive formulation was derived from bond strength tests conducted on various adhesive formulations. Since the adhesive properties of plywood are closely related to the parameters of the hot pressing process, orthogonal experiments were designed to seek for the optimal adhesive preparation process based on the optimal adhesive formulation, which means getting the most appropriate hot-pressing temperature, hot-pressing time, amount of adhesive applied, and mixing time. The design of the orthogonal experiment is shown in Table 2. The stirring temperature of the whole process is 100 °C; the stirring speed is 150 r/min and the plywood samples are made of three-ply veneer.

### 2.5. Preparation of Plywood Samples

**Figure 1:**
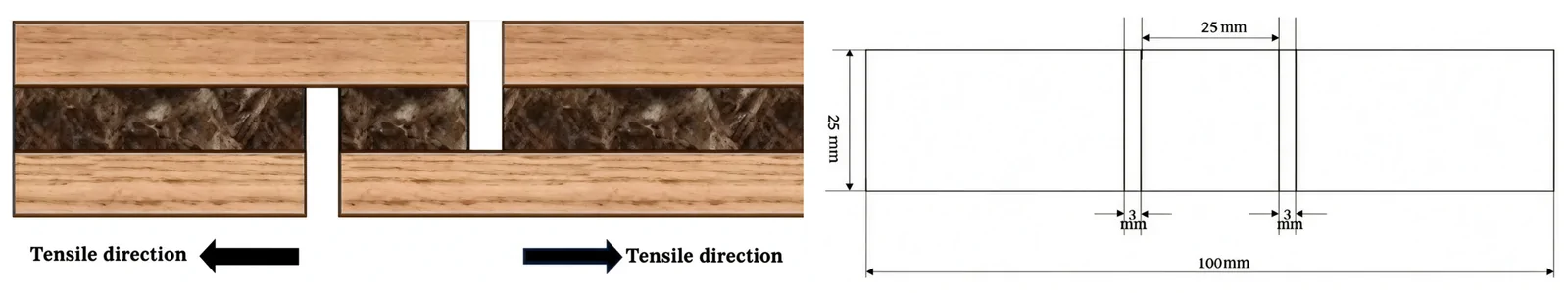
Dimension of plywood samples.

The plywood samples were prepared under the following conditions: adhesive application volume was 280 g/m^2^; the method of adhesive application was one-sided; the plywood samples were made of three-ply veneer; the hot pressing time was 10 min; the hot pressing pressure was 2–3 MPa; and the hot pressing temperature was 130 °C. The conditions in plywood preparation were explored and are shown in Table 2. After hot pressing was completed, the plywood samples were stored at room temperature for at least 24 h, after which the samples were sawn to 100 × 25 mm and then tested. The dimension of plywood samples is shown in Figure 1.

### 2.6. Measurement of Dry- and Wet-Shear Strength

#### 2.6.1. Measurement of Dry Shear Strength

The specimens (100 × 25 mm) were put into a universal mechanical testing machine. The stretching rate was 10 mm/min, and each group was repeated at least 3 times.

#### 2.6.2. Measurement of Wet Shear Strength

The wet shear strength of plywood is determined in accordance with the provisions of the national standard of the People’s Republic of China (GB/T 17657-2022). The specimens were immersed in water at 63 ± 3 °C for 3 h, dried at room temperature for 10 min, and then subjected to a tensile test. The stretching rate was 10 mm/min, and each group was repeated at least 3 times.

### 2.7. Measurement of Fourier Transform Infrared Spectroscopy (FTIR)

FTIR measurements were conducted by directly applying ESO and CLS on KBr. The CLS-ESO and CLS-MA-ESO adhesives were cured in a forced air drying oven until the weight remained constant, and the temperature of the forced air drying oven was set based on the hot pressing temperature to replicate changes in the adhesives after hot pressing. FTIR spectra of various cured adhesives were recorded using a Spectrum 65 spectrometer (Perkin Elmer Co., Ltd., Springfield, IL, USA) with a 4 cm^−1^ resolution and 32 scans within the 500 to 4000 cm^−1^ range [31].

### 2.8. Measurement of Differential Scanning Calorimetry (DSC)

Sample 1, 5–10 mg of ESO, was taken in the crucible for testing. Sample 2 was prepared by mixing MA with ESO in a certain proportion and adding a certain proportion of CLS after mixing uniformly at 100 °C and stirring for 3 min. Sample 3 was prepared by mixing CLS with ESO in a certain proportion and stirring for 3 min at 100 °C. Then, these three samples were taken at 5–10 mg respectively and put into the sample cell of the DSC-4000 differential scanning calorimetry tester (Perkin Elmer Co., Ltd., US) to perform tests. Nitrogen was used as the protective gas, the heating rate was 5 °C/min, and the program temperature was from 0 °C to 230 °C [28].

### 2.9. Thermogravimetric Analysis (TG/DTG)

First, 5–10 mg of ESO was taken in a crucible for testing. The CLS-ESO and CLS-MA-ESO adhesives were cured in a forced air drying oven until the weight was constant, and the temperature of the forced air drying oven was based on the hot pressing temperature to replicate the changes of the adhesives after hot pressing. The resulting samples were then tested in the HCT-4 microcomputer differential thermal balance (Heng Jiu Experimental Equipment, Beijing, China). Nitrogen was used as the protective gas with a flow rate of 60 mL/min, and the temperature was increased from room temperature to 700 °C at a rate of 10 °C/min [32].

## 3. Results and Discussion

### 3.1. Measurement of Adhesive Strength

#### 3.1.1. Results of a Controlled Experiment in Shear Strength

**Figure 2:**
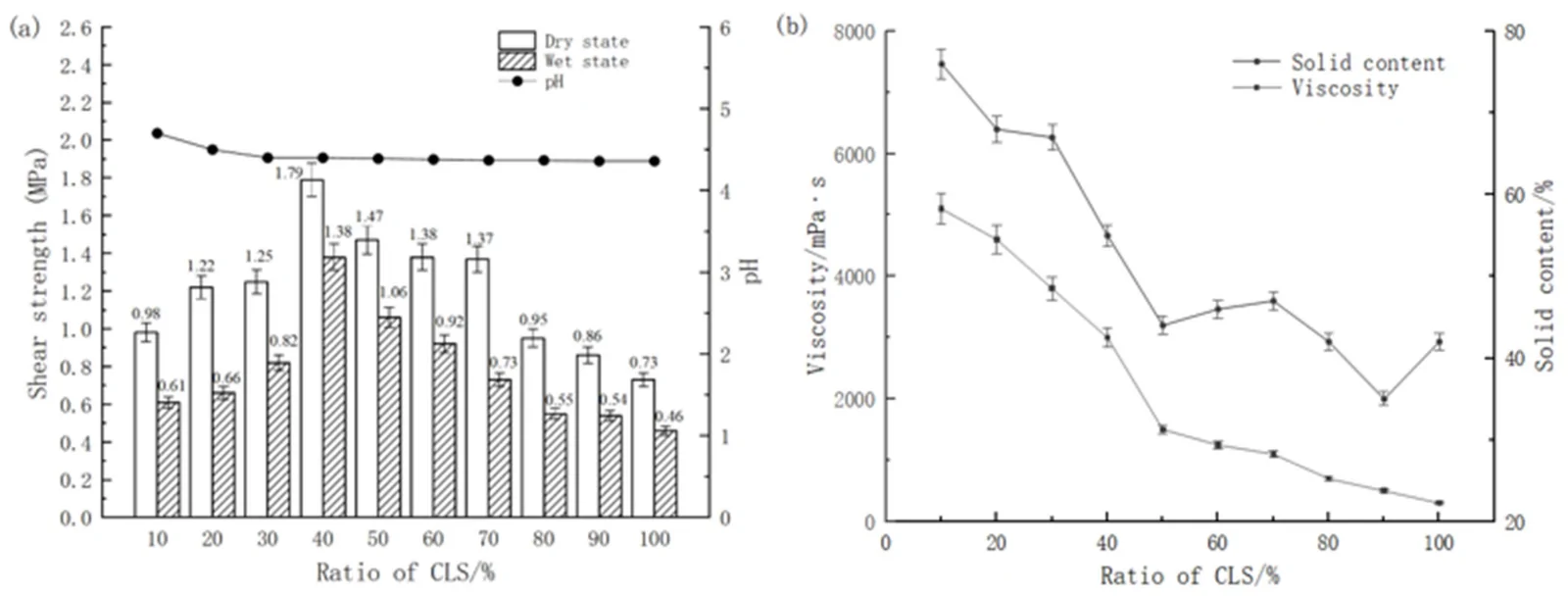
(**a**) Effect of CLS substitution ratio on adhesive shear strength and pH (pH measured at 25 ± 1 °C). (**b**) Effect of CLS substitution ratio on viscosity and solid content of adhesives at 23 ± 1 °C.

The adhesive strength and pH test results of ESO are shown in Figure 2a. The adhesives’ shear strength increased initially, then decreased as the CLS substitution rate in the curing agent grew from 10% to 100%. However, there was little effect on the pH of the adhesive. In the controlled experiment, the adhesive that delivered the highest performance had a CLS substitution rate of 40%. When the CLS substitution ratio exceeded the optimal value of 40 mol%, the shear strength decreased sharply. Combined with existing research, the performance deterioration is mainly attributed to three factors: phase separation, plasticization effect, and residual unreacted carboxyl defects. Excessive high-polarity CLS exhibits poor compatibility with hydrophobic ESO-MA crosslinked matrix, leading to molecular aggregation and microphase separation inside the adhesive layer, forming microscopic void defects that damage the integrity of the crosslinking network. Meanwhile, the redundant CLS that fails to participate in the esterification crosslinking reaction disperses in the network and may acts as a plasticizer, increasing the distance between molecular chains and reducing the cohesive strength of the adhesive film. In addition, maleic anhydride is insufficient to consume all carboxyl groups of excess CLS, plenty of free hydrophilic -COOH groups may remain in the system. These groups easily absorb moisture and form weak bonding interfaces, further reducing dry and wet shear strength [33,34,35]. The results of ESO viscosity and solid content tests are shown in Figure 2b. As the ratio of CLS increased, the viscosity and solid content of the adhesive decreased, which may be due to the large molecular weight of CLS; adding too much CLS will hinder the cross-linking reaction, so the addition of CLS should not be too much.

#### 3.1.2. Results of Orthogonal Experiment in Shear Strength

Table 3 demonstrates the orthogonal results of ESO shear strength under different preparation processes. The influencing order of various factors on wet shear strength is hot pressing temperature > mixing time > hot pressing duration > glue spread. The optimal combination of factor levels was A2B1C2D3: hot-pressing temperature 130 °C, hot-pressing time 10 min, glue spread 280 g/m^2^, and mixing time 70 min. The measured wet shear strength of this optimal experimental group was 1.232 MPa.

As presented in Table 4, variance analysis indicates that hot pressing temperature and mixing time have significant influences on plywood wet shear strength (*p* < 0.05). Further independent validation experiments will be carried out in subsequent research to verify the stability of the optimized preparation process.

### 3.2. Analysis of FTIR

**Figure 3:**
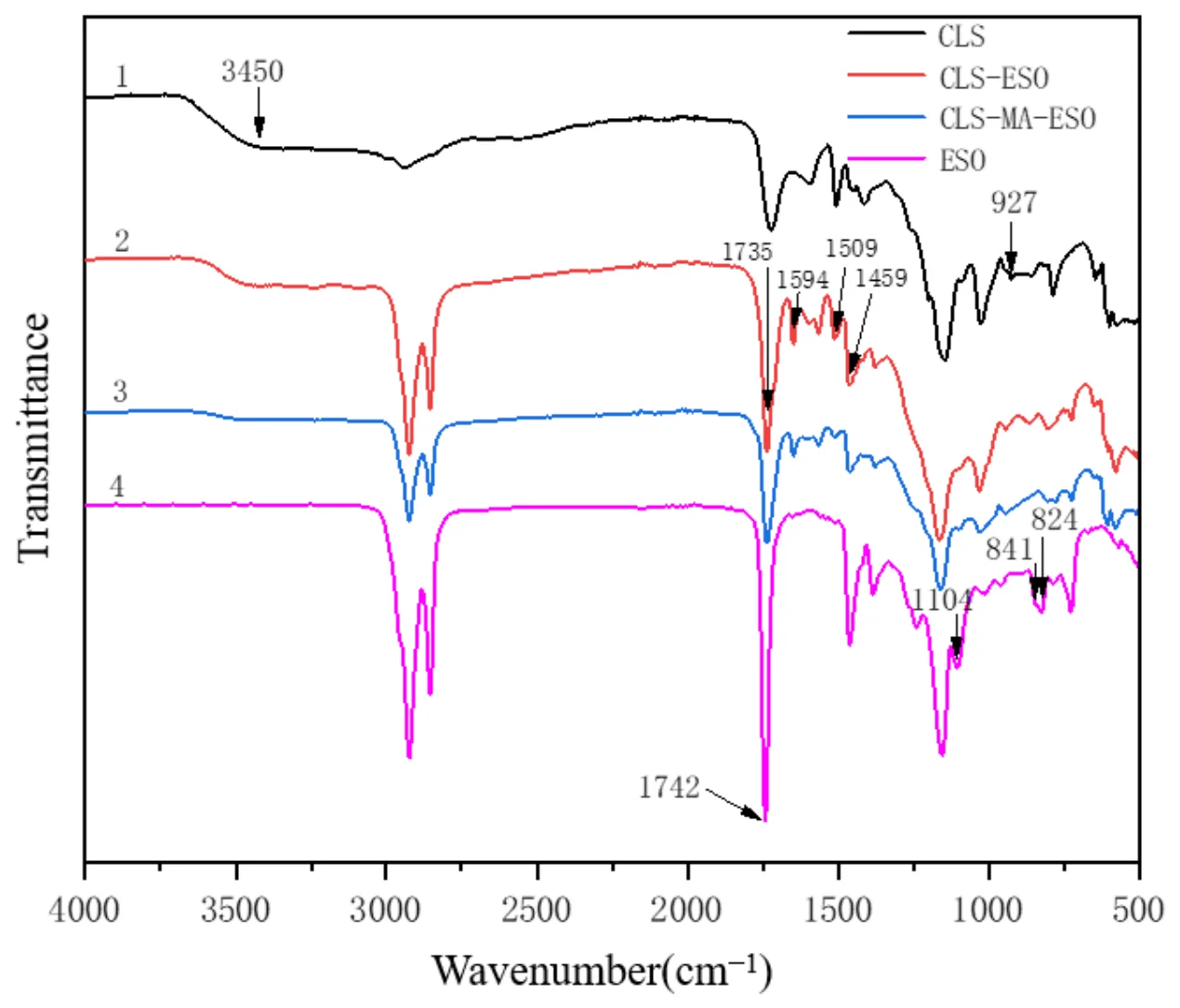
FTIR of various adhesive raw materials and different curing agents after curing.

Figure 3 shows the FTIR of various adhesive raw materials and different curing agents after curing. In the FTIR spectrum, the absorption peaks at 824 cm^−1^ and 841 cm^−1^ are assigned to oxirane (epoxy) rings of ESO, and the band at 1104 cm^−1^ corresponds to aliphatic C-O-C ether stretching vibration. The peak at 890 cm^−1^ belongs to cyclic anhydride of maleic anhydride. The band at 927 cm^−1^ is attributed to the O-H out-of-plane bending vibration of carboxylic acid dimers. The characteristic aromatic C=C skeletal vibration peaks of lignin segments appear at 1594 cm^−1^, 1509 cm^−1^ and 1459 cm^−1^. The strong absorption at 1742 cm^−1^ is the C=O stretching vibration of ester groups in the ESO triglyceride backbone. Two typical carbonyl stretching bands of cyclic maleic anhydride are observed at 1775 cm^−1^ and 1853 cm^−1^. The broad band near 3450 cm^−1^ corresponds to O-H stretching vibration of hydroxyl and carboxyl groups [36,37,38].

**Figure 4:**
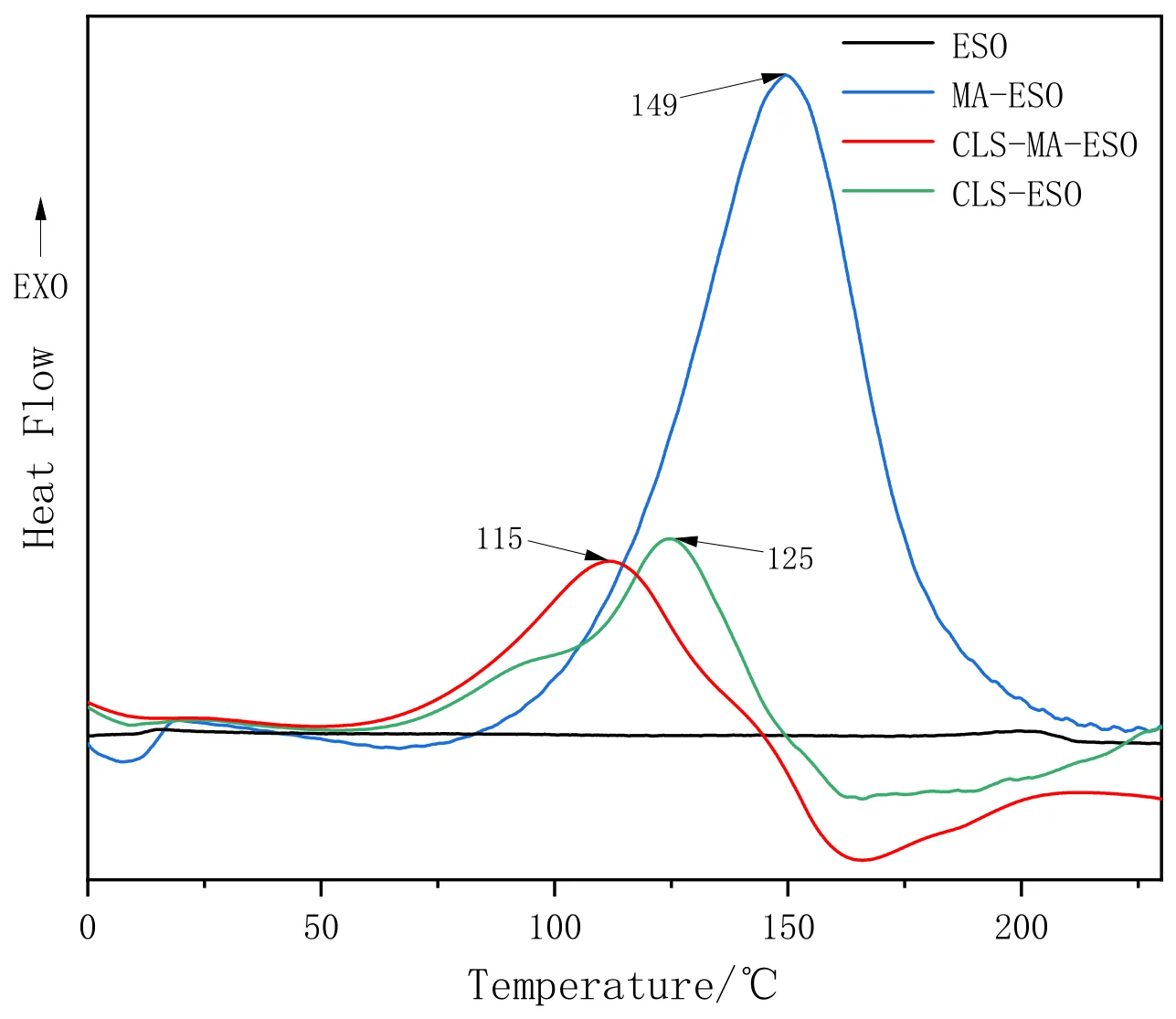
DSC of adhesive raw material and curing processes with different curing agents (heating rate: 5 °C/min).

A schematic diagram of the curing process of the adhesive system is shown in Figure 4. Compared with the spectrum of pure ESO, the epoxy characteristic absorption peaks at 824 cm^−1^ and 841 cm^−1^ of CLS-ESO and CLS-MA-ESO samples were significantly weakened instead of completely disappearing; the absorption band at 1104 cm^−1^ corresponding to aliphatic C-O-C ether skeleton remained basically unchanged owing to its inert property in crosslinking reaction. Meanwhile, the -OH stretching vibration peak at 3450 cm^−1^ appeared newly, and the ester carbonyl peak shifted from 1742 cm^−1^ to 1732 cm^−1^. The above phenomena demonstrate that carboxyl groups on CLS underwent ring-opening addition reaction with epoxy groups of ESO, and further generated hydroxyl-containing crosslinked networks via esterification reaction [39].

In contrast to the spectrum of pristine CLS, the carboxylic acid O-H bending vibration peak at 927 cm^−1^ disappeared in CLS-ESO and CLS-MA-ESO systems. The carbonyl absorption peak at 1732 cm^−1^ exhibited no obvious displacement between the two groups, verifying that esterification reaction took place between CLS and ESO. Further, the formed crosslinked network did not form abundant hydrogen bonds [40].

The 890 cm^−1^ cyclic anhydride peak of MA weakened markedly in the CLS-MA-ESO composite. Combined with the attenuation of characteristic anhydride carbonyl peaks at 1775 cm^−1^ and 1853 cm^−1^, it is confirmed that maleic anhydride participated in ring-opening crosslinking and induced the migration of carbonyl absorption peaks.

**Scheme 1:**
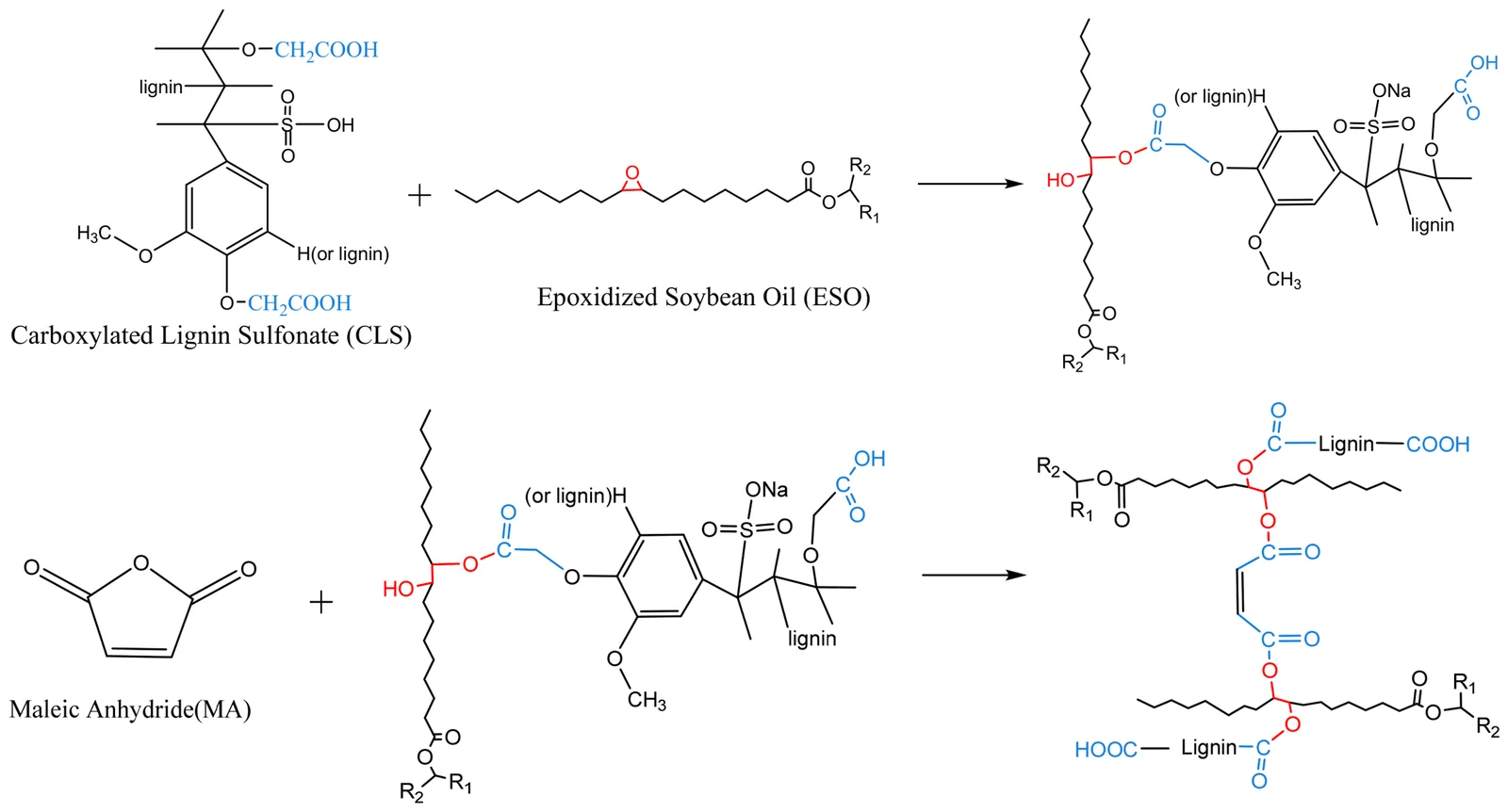
Proposed curing mechanism for the CLS-MA-ESO bio-based adhesive, including ring-opening esterification between carboxylated lignin sulfonate (CLS) and epoxidized soybean oil (ESO), as well as the co-crosslinking effect induced by maleic anhydride (MA).

The aromatic benzene ring C=C skeleton vibration peaks at 1594 cm^−1^, 1509 cm^−1^, and 1459 cm^−1^ of CLS-MA-ESO were lower in intensity than those of CLS-ESO, which indicates that MA reacted with the CLS-ESO intermediate network and reduced the vibration intensity of benzene ring structures. Consistent with the findings of the previous research [25], MA can also conduct esterification crosslinking with ESO, jointly leading to the reduction in absorbance intensity of benzene ring-related peaks (Scheme 1).

### 3.3. Analysis of DSC

Figure 4 shows the DSC curing diagrams of adhesive raw materials and different curing agents. As can be seen from the figure, they have different DSC exothermic curves. There is no exothermic peak for ESO when there is only ESO raw material, which proves that the curing reaction cannot occur by heating ESO. The exothermic peak of ESO cured by only the MA curing agent was 149 °C, but it decreased to 115 °C after adding CLS. This phenomenon can be explained by the fact that CLS reduces the activation energy of the reaction when MA cures ESO. Lu et al. also saw this temperature change in a similar epoxy anhydride system [41]. The exothermic peak occurs at 125 °C when ESO is cured with a single CLS curing agent. He et al. found a similar phenomenon in their study and attributed it to the occurrence of an esterification reaction, which usually occurs at 100–170 °C. This proves that CLS forms a crosslinked network with ESO through covalent bonding [29]. From the DSC results, it can be concluded that the CLS-MA-ESO adhesive can undergo curing at around 115 °C. Due to the moisture content of the adhesive, rapid curing necessitates higher temperatures. Furthermore, the reaction between ESO and the wood surface requires higher temperatures [42].

### 3.4. Analysis of TG/DTG

**Figure 5:**
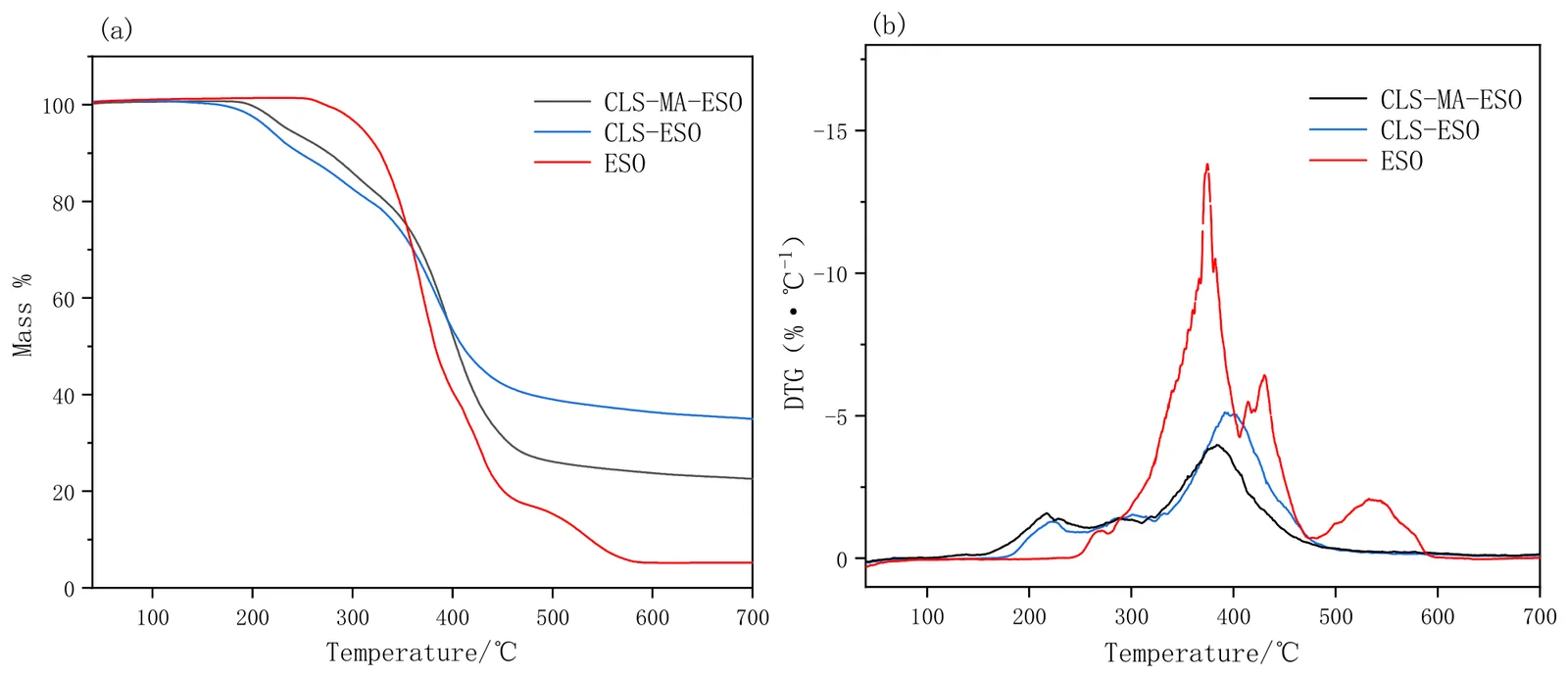
TG and DTG curves of raw adhesive materials and cured adhesives with different curing agents: (**a**) TG curves; (**b**) DTG curves: 10 °C/min under nitrogen atmosphere.

Thermogravimetric analysis was carried out for pure ESO, cured CLS-ESO, and ternary cured CLS-MA-ESO samples, as displayed in Figure 5. Pure ESO started thermal decomposition at 280 °C, with major degradation spanning 280–475 °C, which originated from the thermal cleavage of intrinsic ester chains in soybean oil [39].

CLS-ESO and CLS-MA-ESO exhibited two-stage thermal degradation profiles with similar overall trends. The first low-temperature degradation region corresponds to the decomposition of lignosulfonate fractions [43]. CLS-ESO initiated mass loss at 160 °C (160–260 °C), while CLS-MA-ESO showed a slightly higher onset temperature of 175 °C (175–240 °C). Although the starting temperature of this low-temperature stage of CLS-MA-ESO is marginally higher, partial lightweight small-molecule fragments formed by MA crosslinking degrade within this interval. Nevertheless, both onset temperatures (160 °C, 175 °C) are distinctly higher than the practical hot-pressing processing temperature of 130 °C, proving no thermal degradation occurs during hot-pressing fabrication.

The second degradation stage relates to the rupture of crosslinked ester networks derived from epoxy ring-opening reaction [44]. CLS-ESO and CLS-MA-ESO began intensive decomposition at 320 °C and 330 °C respectively over the range of 320–490 °C.

The 5% mass loss temperature (T_5_%, generally adopted as the comprehensive index evaluating overall thermal stability) of CLS-ESO and CLS-MA-ESO was 216 °C and 235 °C. In terms of comprehensive thermal stability represented by T_5_% and the high-temperature decomposition threshold, introducing MA elevates crosslinking density of the three-dimensional network, thus improving the long-term thermal resistance of the cured adhesive matrix.

Even considering the low-temperature lignosulfonate decomposition stage, both materials remain thermally stable at the forming temperature of 130 °C, far below their low-temperature degradation onset points. Both adhesives possess favorable thermal stability as their T_5_% values exceed 200 °C. Combined with DSC curing data, moderately raising hot-pressing temperature will not trigger thermal degradation of the adhesive system, so appropriate shortening of hot-pressing time is feasible.

## 4. Conclusions

The single-factor controlled experiment illustrated that the dry and wet shear strength of the prepared adhesive presented an obvious rising trend as the dosage of CLS increased within the range below 40 mol%. Strength began to decline once the CLS substitution dosage exceeded 40 mol%. Accordingly, shear strength can be optimized by adjusting CLS content to the optimal dosage rather than simply raising its content continuously. Excessively high loading of CLS would impair the interfacial bonding performance and reduce final bonding strength.

At the optimum CLS dosage of 40 mol%, the plywood attained a maximum dry shear strength of 1.79 MPa and wet shear strength of 1.38 MPa, which far satisfies the requirement of Chinese national standard (≥0.7 MPa) for plywood industrial utilization. Furthermore, orthogonal tests determined the comprehensive optimum preparation parameters: hot-pressing temperature 130 °C, hot-pressing duration 10 min, glue spread 280 g/m^2^, mixing time 70 min.

Combined characterization results of FTIR, DSC, and TG draw the following conclusions. FTIR spectra verified that esterification served as the dominant curing reaction in both CLS-ESO and CLS-MA-ESO systems. The characteristic absorption peaks of epoxy rings were markedly weakened after ring-opening crosslinking instead of nearly disappearing completely; most epoxy functional groups participated in curing reactions to form dense, stable crosslinked networks inside the adhesive matrix.

DSC data manifested that the exothermic curing peak temperature of CLS-MA-ESO was lower than that of pure MA-ESO system. It proves that CLS acts as a catalytic component to lower curing activation energy, which is beneficial to decrease required curing temperature and practical hot-pressing temperature.

TGA tests showed that CLS-MA-ESO possessed a higher initial thermal decomposition temperature than CLS-ESO, confirming that the incorporation of MA increases crosslink density and improves the structural stability and thermal resistance of cured adhesive.

## Figures and Tables

**Table 1:** Formulations of bio-based wood adhesives.

Ratio of CLS/%	ESO/g	CLS/g	MA/g
10%	9.5098	2.5738	2.3164
20%	8.1920	4.4343	1.7737
30%	7.1950	5.8419	1.3631
40%	6.4143	6.9441	1.0416
50%	5.7865	7.8305	0.7830
60%	5.2706	8.5588	0.5706
70%	4.8391	9.1679	0.3929
80%	4.4730	9.6849	0.2421
90%	4.1584	10.1291	0.1125
100%	3.8851	10.5149	0.0000

**Table 2:** Design of orthogonal experiment.

Number	Hot-Pressing Temperatures (°C)	Hot-Pressing Time (min)	Amount of Adhesive Applied (g/m^2^)	Mixing Time (min)
1	120	10	240	30
2	130	20	280	50
3	140	30	320	70
4	150	40	360	90

**Table 3:** The results and range analysis of orthogonal experiments.

Number	Hot-Pressing Temperatures (°C)	Hot-Pressing Time (min)	Amount of Adhesive Applied (g/m^2^)	Mixing Time (min)	Wet Shear Strength/MPa
1	1	1	1	1	0.640
2	3	3	1	3	0.805
3	4	4	1	4	0.656
4	2	2	1	2	0.949
5	2	4	3	1	1.008
6	4	3	2	1	0.620
7	3	2	4	1	0.776
8	1	4	4	3	0.843
9	4	1	4	2	0.648
10	1	3	3	2	0.443
11	2	3	4	4	0.768
12	2	1	2	3	1.232
13	3	1	3	4	0.760
14	3	4	2	2	0.549
15	4	2	3	3	0.939
16	1	2	2	4	0.304
*k*_1_	0.792	1.043	0.996	0.965	-
*k*_2_	1.237	0.977	0.914	0.890	-
*k*_3_	0.959	0.906	1.027	1.208	-
*k*_4_	0.952	1.014	1.003	0.877	-
*R*	0.445	0.137	0.113	0.331	

**Table 4:** The variance analysis of orthogonal experiments on wet shear strength.

Factors	Sum of Squares of Deviations (DEVSQ)	Degree of Freedom (DOF)	F-Value	*p*-Value
Hot-pressing temperatures	0.385	3	14.5	0.027
Hot-pressing time	0.054	3	2.0	0.289
Amount of adhesive applied	0.028	3	1.1	0.482
Mixing time	0.276	3	10.4	0.043
Error	0.026	3		

## Data Availability

The original contributions presented in this study are included in the article. Further inquiries can be directed to the corresponding author.
